# Supplementary material for: Single-cell chromatin accessibility profiling of acute myeloid leukemia reveals heterogeneous lineage composition upon therapy-resistance
Source: Commun Biol. 2023 Jul 21;6:765. doi: 10.1038/s42003-023-05120-6 (PMC10362028; doi:10.1038/s42003-023-05120-6)
Supplement: Supplementary file 2 — Supplementary File [file 42003_2023_5120_MOESM2_ESM.pdf]

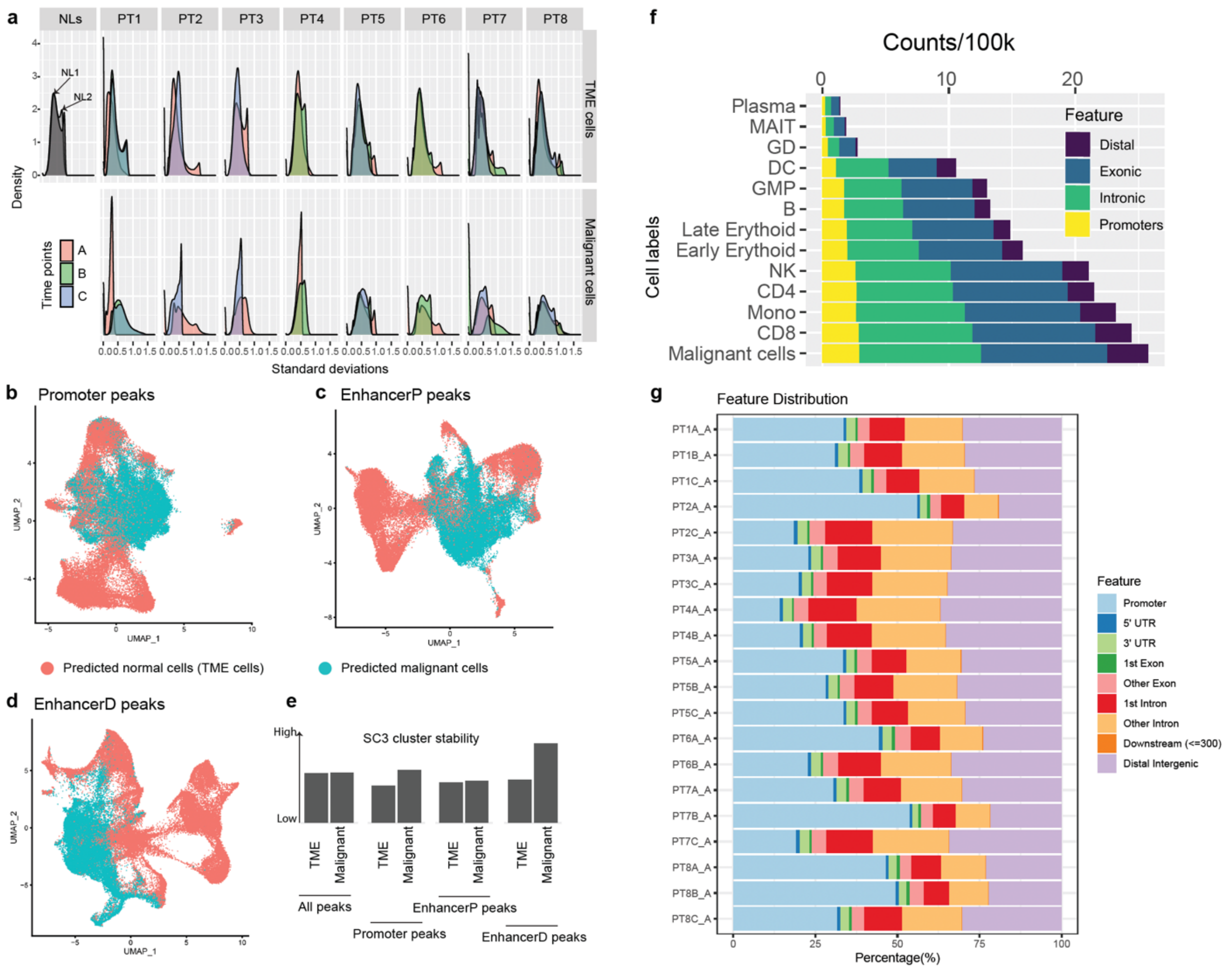

***Supplementary Figure 1. Overview of the scATAC-seq data**. a) Density plots showing open chromatin variations across different patients at different timepoints. UMAP showing predicted malignant cells and TME cells using different set of peaks, such as promoter peak set (b), proximal enhancer peak set (c) and distal enhancer peak set (d). e) SC3 clustering stability using different sets of open chromatin peaks, when considering two major clusters of tumor microenvironment (TME) and malignant cells. f) Barchart showing the number in 100k unit of open chromatin peaks per cell type. g) Genomic annotations of peaks per sample.*

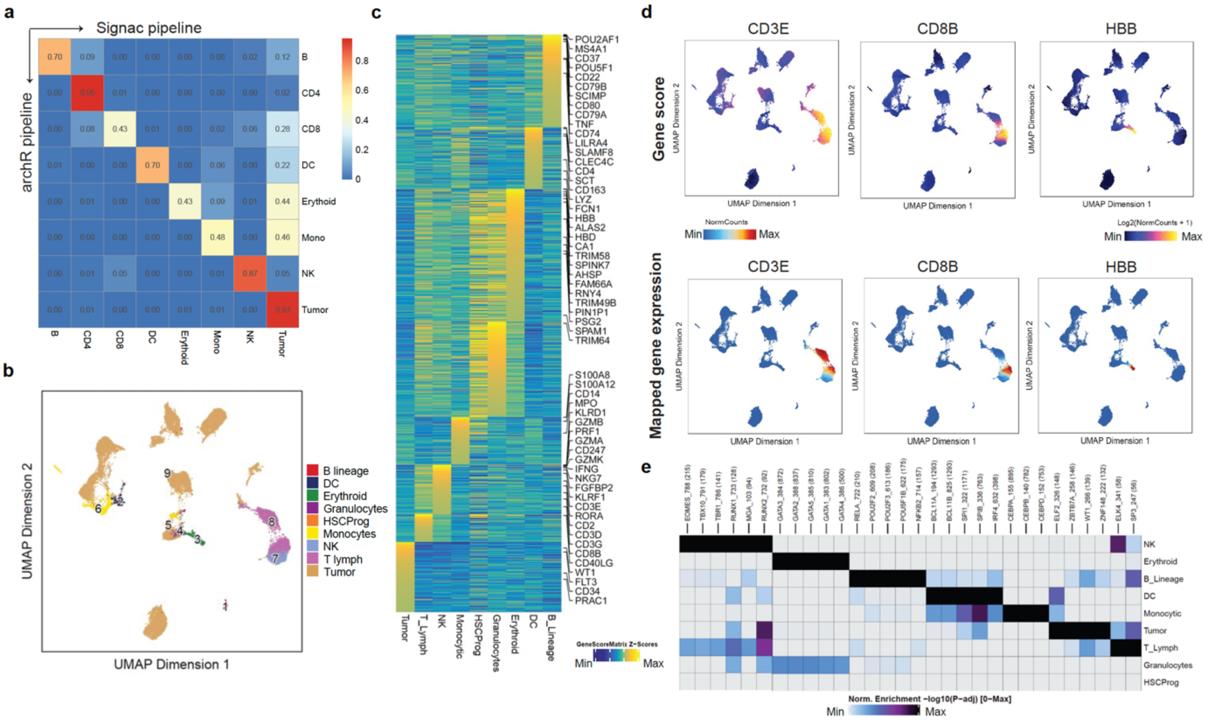

***Supplementary Figure 2. Verification of cell label transfer**. a) Heatmap showing the consistency of major cell type calling between two different scATAC-seq processing pipelines. Percentage of overlapped cell type are labeled on the heatmap. b) UMAP of scATAC-seq cell types predicted using archR pipeline. c) Heatmap showing the marker genes (based on gene activity scores) across different cell types. d) UMAP embeddings of all cells showing mapped gene expression by integrating scRNA-seq data (upper panel), and gene activity scores predicted using scATAC-seq data (lower panel) of CD3E, CD8B and HBB. e) Heatmap showing the motif enrichment across different cell types.*

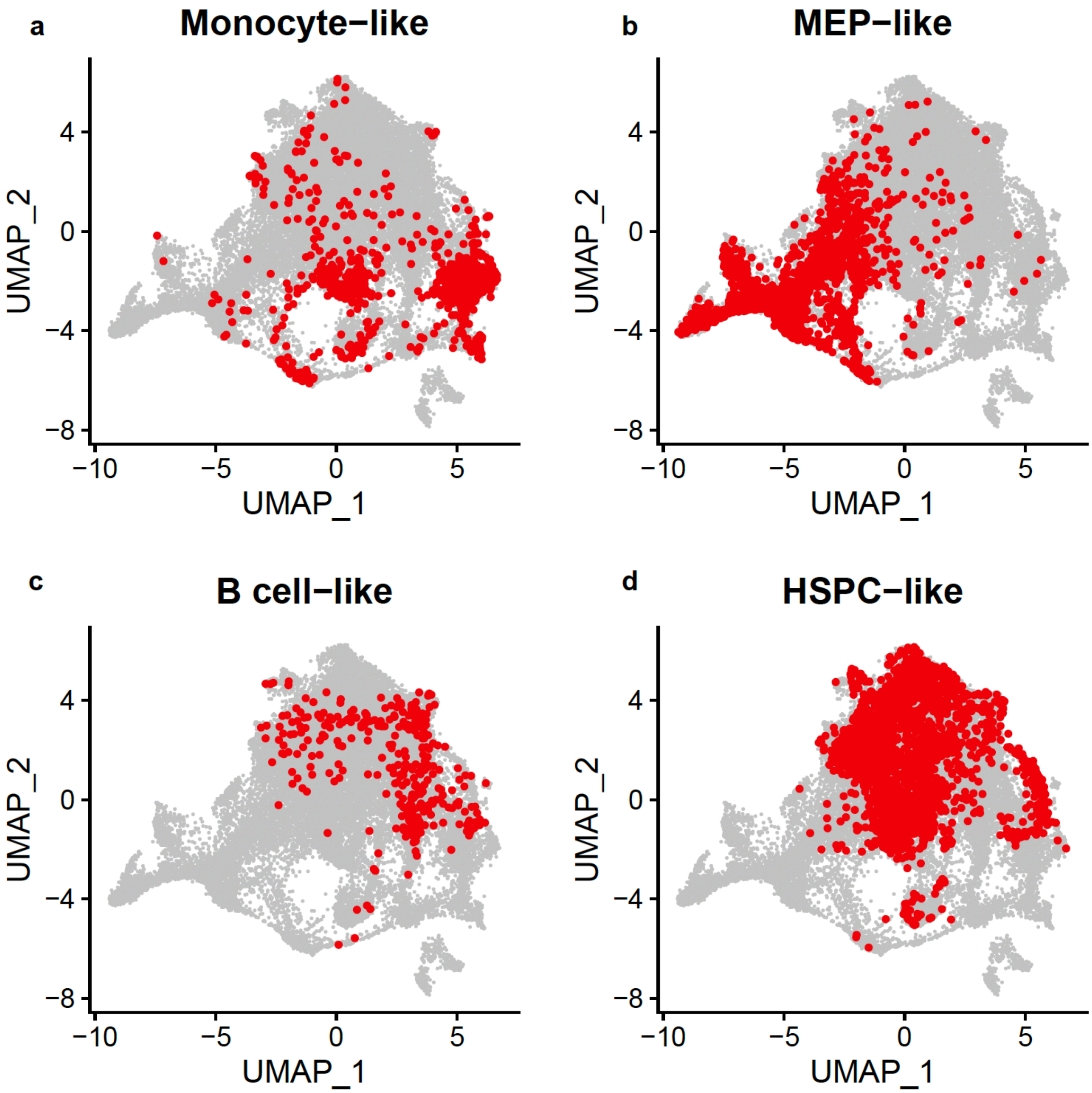

Supplementary Figure 3. UMAPs of all the malignant cells highlighted by cells with cell states assigned. *UMAP embeddings with monocyte-like (a), MEP-like (b), B cell-like (c), and HSPC-like (d) cell states highlighted.*

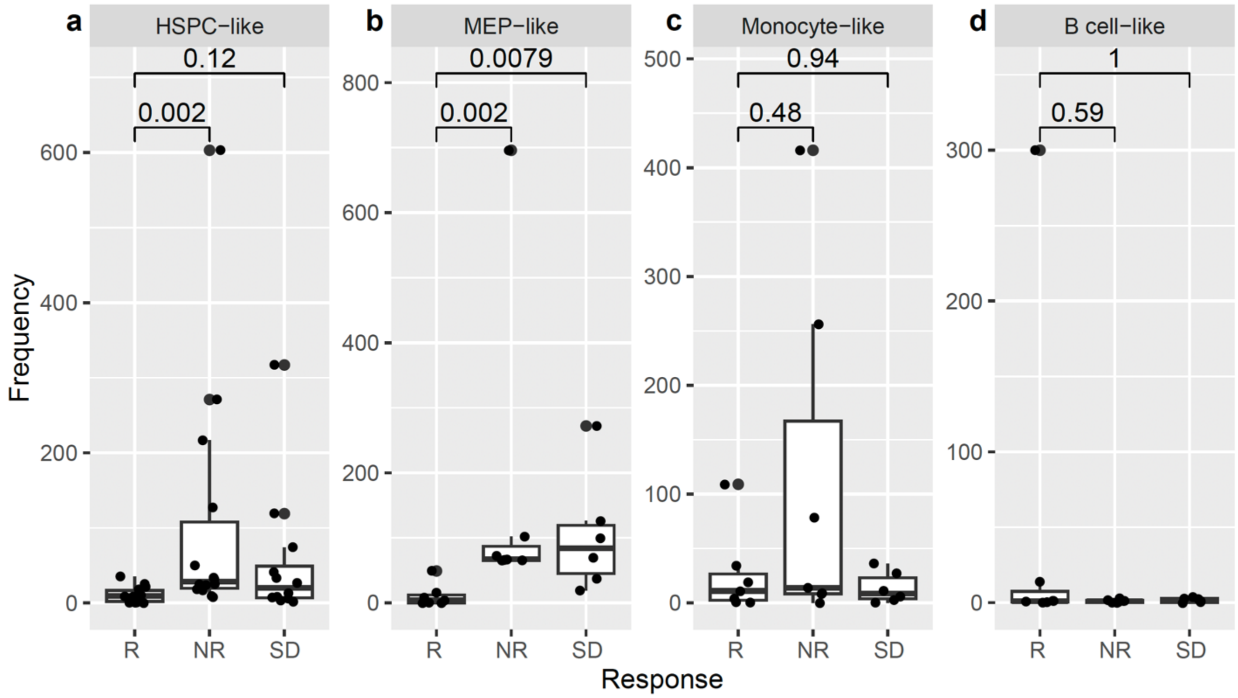

Supplementary Figure 4. Boxplots showing frequencies of each malignant cell state in patient groups of R (Responders), NR (None responders), and SD (Stable disease). *a) HSPC-like cell state. b) MEP-like cell state. c) Monocyte-like cell state. d) B cell-like cell state.*

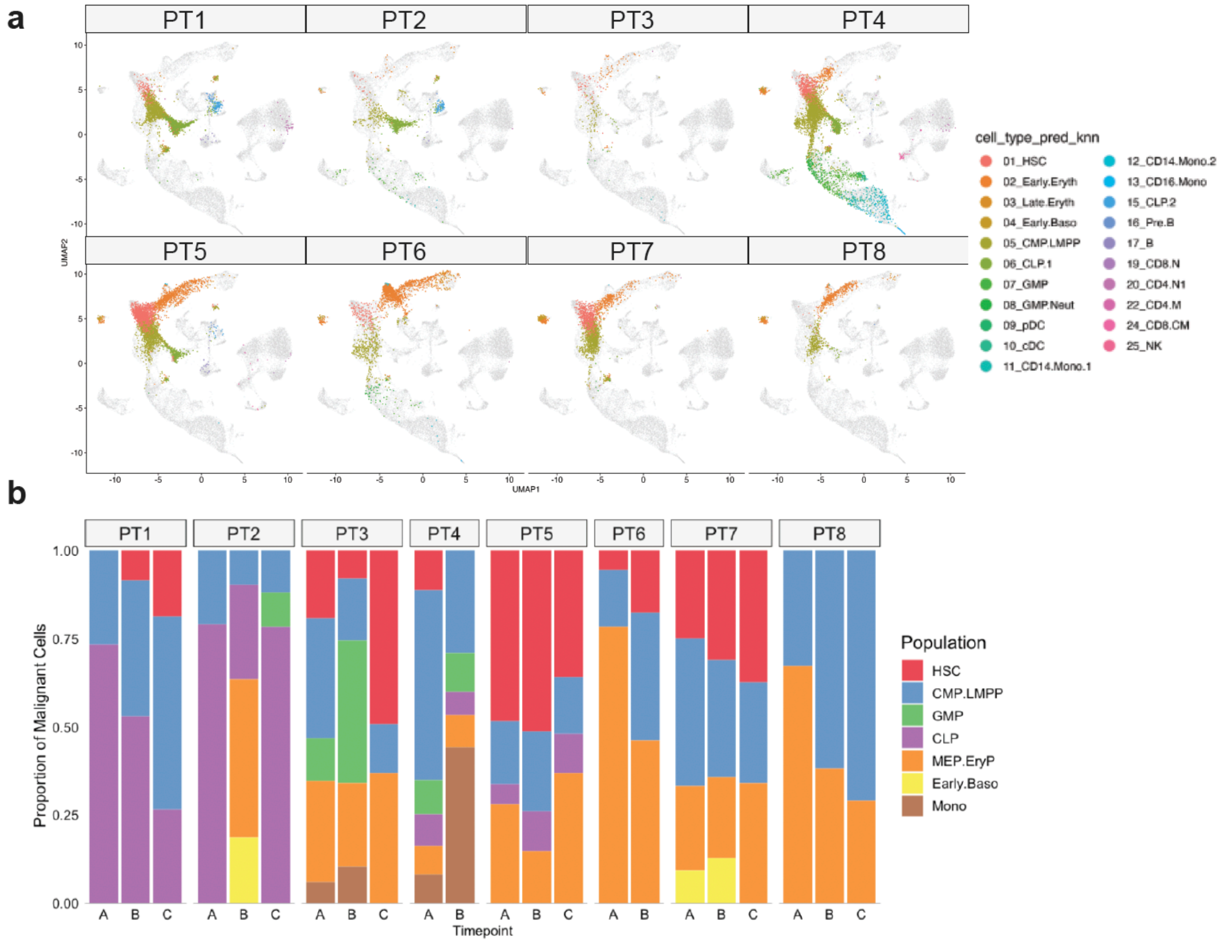

Supplementary Figure 5. Cellular composition validation using scRNA-seq data. *a) Symphony projection of scRNA-seq profiled AML malignant cells onto well-annotated reference. b) Proportion of malignant AML cell states calculated using scRNA-seq data with broad cell class.*

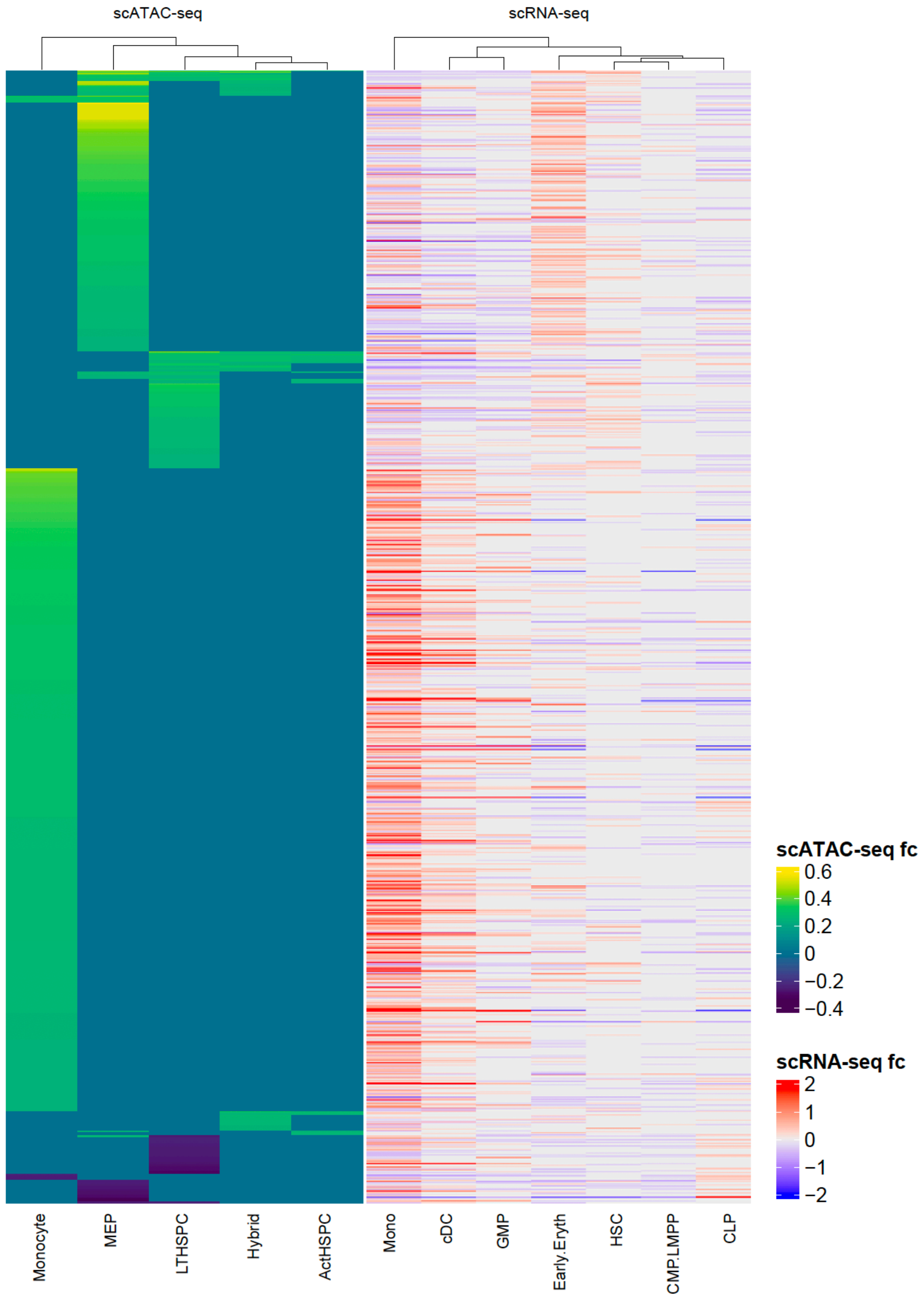

Supplementary Figure 6. Heatmap showing fold changes for DARs using scATAC-seq data (left) and DEGs using scRNA-seq data (right). *Differential events are calculated via comparing AML malignant cells with diverse cell states to the ones without any cell states assigned in both types of data.*

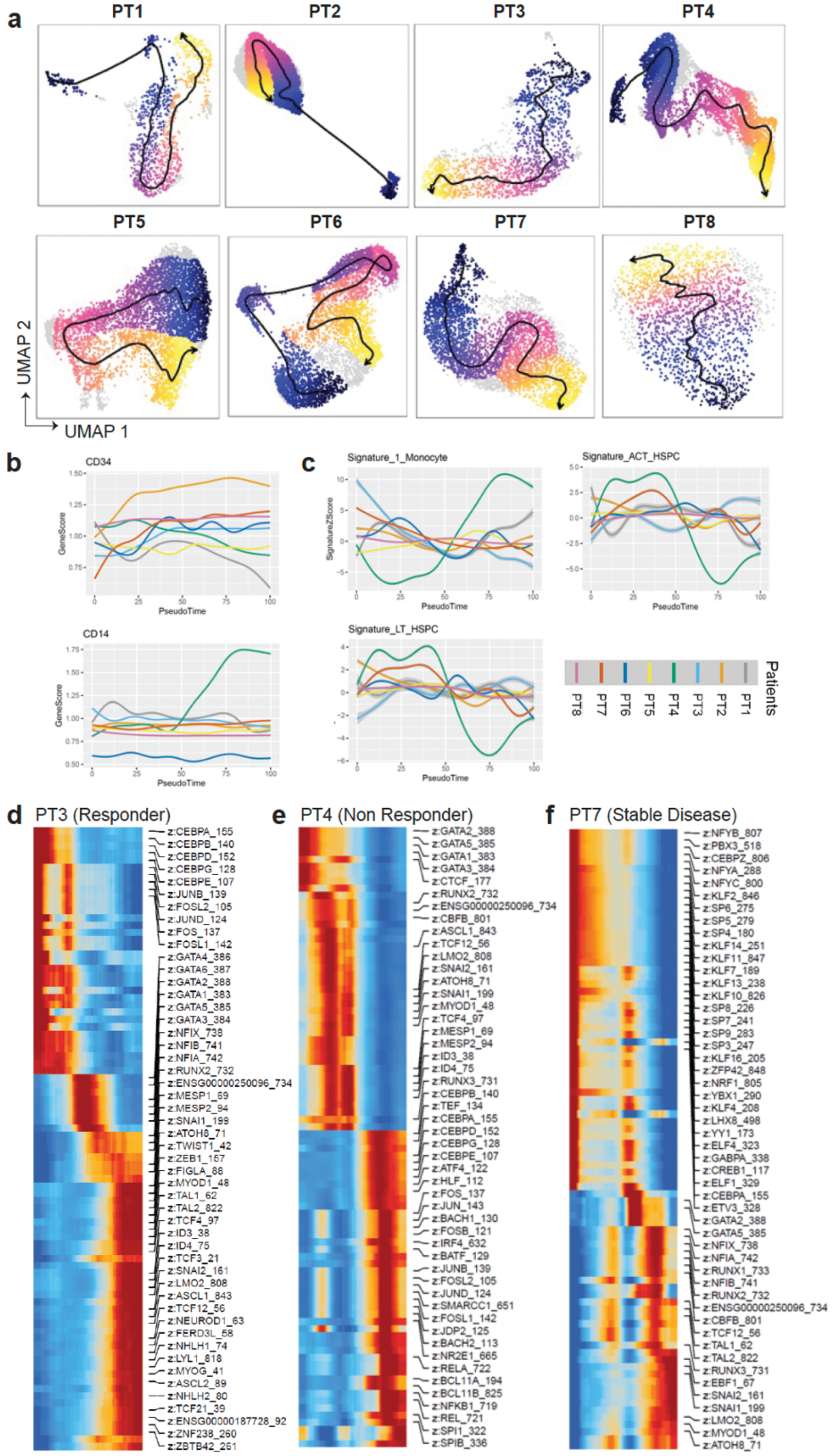

Supplementary Figure 7. Pseudotemporal analysis reveals chromatin accessibility of canonical transcription factor motifs. *a) UMAP embeddings showing the trajectories of all 8 patients. b) Line plots showing the pseudotemporal accessibility changes (based on gene scores) of genes associated with stemness (CD34) as well as those of lineage commitment related genes such as CD14. c) Line plots showing the pseudotemporal signature Z score changes of various accessibility signatures which was based on sorted well-defined hematopoietic cells from different hematopoietic lineage states. Heatmaps showing the pseudotemporal motif enrichment of key transcriptional factors for PT3 (Responder (d)), PT4 (Non-responder (e)) and PT7 (Stable Disease (f)).*
