# Supplementary material for: Single-cell chromatin accessibility profiling of acute myeloid leukemia reveals heterogeneous lineage composition upon therapy-resistance
Source: Commun Biol. 2023 Jul 21;6:765. doi: 10.1038/s42003-023-05120-6 (PMC10362028; doi:10.1038/s42003-023-05120-6)
Supplement: Supplementary file 3 — Description of Additional Supplementary Files [file 42003_2023_5120_MOESM3_ESM.pdf]

Description of Additional Supplementary Files

File name: Supplementary Data 1
Description: AML cell states-related DARs and their linked genes. Each sheet represents one type of AML cell state. DARs were calculated via comparing AML malignant cells with diverse cell states to the ones without any cell states assigned.

File name: Supplementary Data 2
Description: AML cell states-related DARs-linked gene functions. Each sheet represents one type of AML cell state.

File name: Supplementary Data 3
Description: AML cell states-related DARs and their linked genes. DARs were identified via comparing AML cell states to their corresponding normal counterparts. Each sheet represents one type of AML cell state.
