# Supplementary material for: Single-cell chromatin accessibility profiling of acute myeloid leukemia reveals heterogeneous lineage composition upon therapy-resistance
Source: Commun Biol. 2023 Jul 21;6:765. doi: 10.1038/s42003-023-05120-6 (PMC10362028; doi:10.1038/s42003-023-05120-6)
Supplement: Supplementary file 7 — Reporting Summary [file 42003_2023_5120_MOESM7_ESM.pdf]

nature portfolio

Corresponding author(s): Hussein Abbas

Last updated by author(s): Jul 3, 2023

Reporting Summary

Nature Portfolio wishes to improve the reproducibility of the work that we publish. This form provides structure for consistency and transparency in reporting. For further information on Nature Portfolio policies, see our Editorial Policies and the Editorial Policy Checklist.

Statistics

For all statistical analyses, confirm that the following items are present in the figure legend, table legend, main text, or Methods section.

n/a	Confirmed	
☐	☒	The exact sample size (*n*) for each experimental group/condition, given as a discrete number and unit of measurement
☐	☒	A statement on whether measurements were taken from distinct samples or whether the same sample was measured repeatedly
☐	☒	The statistical test(s) used AND whether they are one- or two-sided<br>*Only common tests should be described solely by name; describe more complex techniques in the Methods section.*
☐	☒	A description of all covariates tested
☐	☒	A description of any assumptions or corrections, such as tests of normality and adjustment for multiple comparisons
☐	☒	A full description of the statistical parameters including central tendency (e.g. means) or other basic estimates (e.g. regression coefficient) AND variation (e.g. standard deviation) or associated estimates of uncertainty (e.g. confidence intervals)
☒	☐	For null hypothesis testing, the test statistic (e.g. F, t, r) with confidence intervals, effect sizes, degrees of freedom and P value noted<br>*Give P values as exact values whenever suitable.*
☒	☐	For Bayesian analysis, information on the choice of priors and Markov chain Monte Carlo settings
☒	☐	For hierarchical and complex designs, identification of the appropriate level for tests and full reporting of outcomes
☒	☐	Estimates of effect sizes (e.g. Cohen's *d*, Pearson's *r*), indicating how they were calculated

Our web collection on statistics for biologists contains articles on many of the points above.

Software and code

Policy information about availability of computer code

Data collection	No software was used in the data collection process.
Data analysis	All data analysis and statistical analysis were performed using R software, with version 4.1.4 or higher.

For manuscripts utilizing custom algorithms or software that are central to the research but not yet described in published literature, software must be made available to editors and reviewers. We strongly encourage code deposition in a community repository (e.g. GitHub). See the Nature Portfolio guidelines for submitting code & software for further information.

Data

Policy information about availability of data

All manuscripts must include a data availability statement. This statement should provide the following information, where applicable:

- Accession codes, unique identifiers, or web links for publicly available datasets
- A description of any restrictions on data availability
- For clinical datasets or third party data, please ensure that the statement adheres to our policy

All raw data have been uploaded to European Genome-Phenome Archive (EGA) (Accession # EGAD00001007675) and will be publicly released upon manuscript acceptance. An access code for reviewers can also be provided upon request.

Human research participants

Policy information about studies involving human research participants and Sex and Gender in Research.

Reporting on sex and gender	No sex and gender information was reported. No sex and gender-related analysis was conducted either.
Population characteristics	past and current diagnosis and treatment information were collected and included in the study.
Recruitment	Bone marrows from eight adult (>=18 years of age) patients on NCT02397720 protocol were included in this study. All patients had histologically proved relapsed or refractory acute myeloid leukemia. Timepoint A was collected at time of proven relapse or primary refractoriness. Timepoints B and C were collected during the treatment with azacitidine 75 mg/m2 (administered intravenously on days 1 to 7 over 60 to 90 minutes or subcutaneously), and nivolumab 3mg/kg (administered as a 60 to 90 minutes intravenous infusion) on days 1 and 14 of each cycle treated on NCT02397720 protocol. Written informed consent was obtained from all participants. The study was conducted in accordance with the Declaration of Helsinki and had IRB approval.
Ethics oversight	Written informed consent was obtained from all participants. The study was conducted in accordance with the Declaration of Helsinki and had IRB approval.

Note that full information on the approval of the study protocol must also be provided in the manuscript.

Field-specific reporting

Please select the one below that is the best fit for your research. If you are not sure, read the appropriate sections before making your selection.

☒ Life sciences ☐ Behavioural & social sciences ☐ Ecological, evolutionary & environmental sciences

For a reference copy of the document with all sections, see nature.com/documents/nr-reporting-summary-flat.pdf

Life sciences study design

All studies must disclose on these points even when the disclosure is negative.

Sample size	No sample-size calculation was conducted.
Data exclusions	Samples were removed during data analysis step due to insufficient capture of enough single cells from patients.
Replication	Sequencing data were generated from cancer patients at multiple treatment timepoints.
Randomization	Not relevant to our study. Patients with specific diagnosis and treatments are grouped.
Blinding	Not relevant. Supervised group comparisons are needed for our study.

Reporting for specific materials, systems and methods

We require information from authors about some types of materials, experimental systems and methods used in many studies. Here, indicate whether each material, system or method listed is relevant to your study. If you are not sure if a list item applies to your research, read the appropriate section before selecting a response.

Materials & experimental systems

n/a	Involved in the study	
☒	☐	Antibodies
☒	☐	Eukaryotic cell lines
☒	☐	Palaeontology and archaeology
☒	☐	Animals and other organisms
☒	☐	Clinical data
☒	☐	Dual use research of concern

Methods

n/a	Involved in the study	
☒	☐	ChIP-seq
☒	☐	Flow cytometry
☒	☐	MRI-based neuroimaging
